# Phenomenally Large and Small Babies

**Published:** 1889-02-02

**Authors:** 


### BABIES.

(*Continued from p.* 265.)

### V.—PHENOMENALLY LARGE AND SMALL BABIES.

NOTHING decidedly abnormal in the way of babyhood is a very pleasant subject for contemplation, however curious it may be, or however interesting from special points of view. But we are none of us able altogether to ignore the fact that very queer babies are occasionally born, or able altogether to resist a certain curiosity about them, and there are some to whom it may be worth while to devote a chapter.

Among the most interesting and least painful of Nature's freaks have been her tiniest productions, notable among whom was the infant destined to become the great Sir Isaac Newton, who was at one time such a wee specimen of his kind that he was put into a quart pot. What was the starting weight of the future great mathematician does not appear to have been recorded, but, small as he was, no doubt he had a considerable advantage over many, both in point of weight and dimensions. Mr. Cullingworth, formerly surgeon to St. Mary's Hospital, Manchester, has publicly stated that the smallest living baby he ever saw was one which weighed 2 lb , and measured 14 in. in length. But, then, that was four weeks old. In the account of a number of Lilliputian mortals, given before a meeting of the Obstetrical Society, one is spoken of as weighing 1 lb. 13 oz. at about seven weeks old. Dr. Bierbaum, of Dorsten, describes a child he saw which measured 13½ in., and weighed 1½ lb. An American paper, the *Boston Journal*, a year or two back mentioned a child born slightly under this weight. Says the anonymous writer: "We know a bright, intelligent little miss, now residing in this city, who is eleven years old, who weighs about 60 lb., and who, when she opened her eyes upon this world, weighed less than 1½ lb. She was the tiniest piece of humanity which ever we heard of. The nurse in washing and dressing her used to lay her in the palm of her hand, and the first few days of her life were mostly spent wrapped up in cotton wool, and placed in the basket beside the stove to keep her warm. Her head would go into a small-sized tea-cup. Can any one tell of a smaller edition of humanity that lived and grew to be a bright and promising little lady?"

The little Boston lady was decidedly small, but there have been smaller. Dr. Willing, in April, 1874, exhibited to the Obstetrical Society the body of a child that measured 11 in., and weighed 1¼ lb. only. It is true this little creature only lived 44 hours, but she seems to have made vigorous use of her time while she did live, and is said to have cried as lustily as a fully-developed infant; but with all her efforts, supplemented as no doubt they were, by the best the doctors could do for her, she could never get comfortably warm. Even this puny little creature of 1¼ lb. cannot be accounted the smallest on record. Dr. Barker described in the *Medical Times* some fifteen or sixteen years ago a female child which was 11 in. long, and weighed just 1 lb. when born. Poor little Katherine Kelly, a dwarf 34 in. high and proportionately small, exhibited in this country in 1785, gave birth to a child seven times as large as this, and more than twice as long. The unfortunate little mother lived only two hours. The tiny mite mentioned by Dr. Barker lived, and at the age of 4½, though still small, was said to be quite healthy and taking her food with a zest that gave every promise of a satisfactory future.

We cannot find any record of a birth-weight lighter than this; but, if all accounts may be relied upon, there have been children that have measured less than even this 11-in. pigmy. When Stanislaus was king of Poland he had attached to his court a nobleman and his wife, both of ordinary stature, who had had six children, three of whom grew to the ordinary dimensions, while the other three proved dwarfs, and curiously enough, they were born alternately—first a dwarf, and then a child of ordinary dimensions and normal after. growth. The eldest, who lived to be Count Borolowski, is said to have weighed a pound and a-quarter at birth, and to have measured 8 in. in length. He was presented for baptism in a plate, and for some time had a wooden shoe for his cot. His mouth is said to have been too small to permit of his being suckled by his mother, and a goat had to be procured. He grew to be 3 ft. 2 in. in height, and was wont to say that, though he had been small at birth, he was neither weakly nor puny, and certainly in later life he gave every evidence of mental and physical vigour.

Another dwarf has been mentioned in connexion with the court of this unfortunate Polish king. This appears to have been a brother of Count Borolowski. He was 8 or 9 in. long at birth, and grew to be only 33 in. high. He died in 1764, an old man at about three and twenty years of age.

Thus it will be seen that those who have been remarkable for their diminutive proportion at mature age, have often been quite as remarkable at the outset of their careers. But this has not always been the case. The famous Geoffrey Hudson, who was first introduced to the court of Charles I. in a pie at an entertainment given to the Duke of Buckingham, was of ordinary dimensions when born, but he never made much headway afterwards. Readers of Scott will remember that he figures prominently in "Peveril of the Peak," and in one of his "Notes" the Waverly novelist tells us that Hudson at eighteen years of age was but 18 or 20 in. high. The old ladies of Charles the First's time were able to account for this, as old ladies have so often been able to do in similar cases from that time to this. His mother, it appears, was very fond of pickled cucumbers, and at a critical time was very nearly choked with a bit of gherkin. Now gherkins, it should be observed, are a very small fruit as compared with the cucumber, and some thought her being so nearly choked with this smaller kind might, through sympathy and other influences of the imagination, have some effect on the infant. Hudson, as it has been said, was only 18 or 20 in. high at 18 years of age, and Scott adds that he remained at that height till he was 30 years old, and then began to grow again, finally attaining a height of 3 ft. 9 in.

Nanette Stocker, a well-proportioned accomplished little woman, 33 in. high, who was exhibited in London somewhere about the close of the last century, was said to have been an unusually large child when born. On the other hand, the famous Daniel Lambert, who at his death was so large and heavy that the walls of the room in which he died had to be broken away to permit of his being removed, was quite an ordinary child at birth. The ideal Daniel Lambert as an infant was Master Wybrants, "The Modern Hercules," as he was dubbed when exhibited. At four months this precocious young gentleman weighed 39 lb., measured 2 ft. round the body, 15 in. round the thigh, and 8 in. round the arm. Young Wybrants seems to have been a bonny boy from the outset of his career; but another huge specimen of babyhood, shown in Cornhill in 1780, presented nothing special till he was five or six weeks old when he began to develop amazingly, and by 11 months was 3 ft. 3 in. in length, 2 ft. 6 in. round the chest, and 3 ft. 1 in. round the loins; his thigh measured 1 ft. 9 in.; his arm, 11 in. round; and his wrist 9 in. His weight is simply described as "prodigious"—a curious indication of the odd superstition of the time, not wholly dispelled, yet by the way, that to weigh a baby is unlucky. His weight certainly must have been "prodigious," but they dared not pop him into the scale and see exactly what he weighed. This chubby cherub was born at Enfield paper mills in 1779; and, curious to relate, no purveyor of infants' food seems to have claimed the merit of his Herculean proportions.

(*To be concluded.*)

